# Structure of the ParM filament at 8.5 Å resolution

**DOI:** 10.1016/j.jsb.2013.02.010

**Published:** 2013-10

**Authors:** Pananghat Gayathri, Takashi Fujii, Keiichi Namba, Jan Löwe

**Affiliations:** a MRC Laboratory of Molecular Biology, Hills Road, Cambridge CB2 0QH, UK; b Graduate School of Frontier Biosciences, Osaka University, 1-3 Yamadaoka, Suita, Osaka 565-0871, Japan; c Riken Quantitative Biology Centre, 1-3 Yamadaoka, Suita, Osaka 565-0871, Japan

**Keywords:** Bacterial cytoskeleton, Actin, Cytomotive, Dynamic instability

## Abstract

The actin-like protein ParM forms the cytomotive filament of the ParMR*C* system, a type II plasmid segregation system encoded by *Escherichia coli* R1 plasmid. We report an 8.5 Å resolution reconstruction of the ParM filament, obtained using cryo-electron microscopy. Fitting of the 3D density reconstruction with monomeric crystal structures of ParM provides insights into dynamic instability of ParM filaments. The structural analysis suggests that a ParM conformation, corresponding to a metastable state, is held within the filament by intrafilament contacts. This filament conformation of ParM can be attained only from the ATP-bound state, and induces a change in conformation of the bound nucleotide. The structural analysis also provides a rationale for the observed stimulation of hydrolysis upon polymerisation into the filament.

## 1 Introduction

**Fig. 1:**
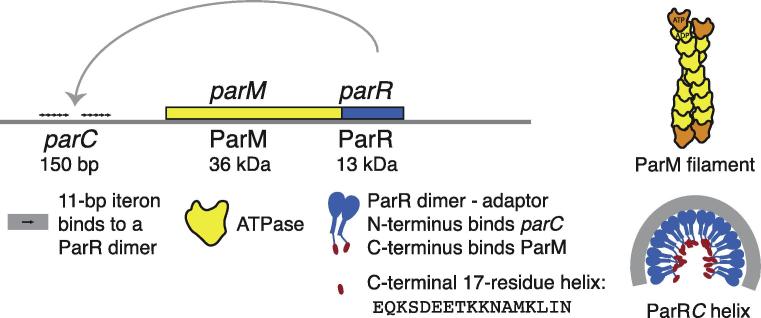
Schematic representation of the components of the ParMR*C* system. The operon for the ParMR*C* system consists of ParM and ParR regulated by the centromeric region of *parC*. ParM is an ATPase of actin fold, and forms double helical filaments. ParR is a repressor of ParM and ParR expression and also the adaptor between the DNA and the filaments. It links the *parC* DNA and the end of ParM filaments. Ten ParR dimers bind to the ten iterons (11-base pairs each) of *parC* and form the ParR*C* helical ring.

ParMR*C*, the eponymous Type II plasmid segregation system, provides a well-documented example of an actin-like double helical filament functioning as a linear motor, by actively pushing R1 plasmids towards the cell poles of *Escherichia coli* (Gerdes et al., 2010; Salje et al., 2010). Hence filaments of the ParM protein have been classified as cytomotive (Löwe and Amos, 2009). The system comprises three components: ParM, a polymerising motor of actin fold, *parC*, a centromeric, repeated DNA sequence on the plasmid and ParR, the adaptor protein that links the filament and *parC* DNA (Fig. 1). The mechanism of action of ParMR*C*, one of the simplest mitotic spindles, has been studied using a combination of techniques involving biochemical and immunofluorescence techniques (Møller-Jensen et al., 2003), X-ray crystallography (van den Ent et al., 2002; Møller-Jensen et al., 2007; Schumacher et al., 2007), fluorescence microscopy both *in vitro* and *in vivo* (Garner et al., 2004, 2007; Campbell and Mullins, 2007), electron microscopy (Orlova et al., 2007; Popp et al., 2008; Galkin et al., 2009), and cryo-electron microscopy of vitreous sections (CEMOVIS) of *E. coli* cells expressing ParM (Salje et al., 2009). These approaches have provided detailed mechanistic insights into the plasmid partitioning mechanism by ParMR*C* [reviews (Gerdes et al., 2010; Salje et al., 2010)] Fig. 1.

ParM forms actin-like double helical filaments comprising two protofilaments. In contrast to actin filaments with a right-handed twist, ParM filaments are left-handed (Orlova et al., 2007; Popp et al., 2008). They also differ from actin in their dynamic behaviour. ParM filaments exhibit dynamic instability, as opposed to the treadmilling properties of actin (Garner et al., 2004), which is thought to be an essential feature facilitating plasmid partitioning. Structural description at atomic resolution, of the various conformational states of the filament and monomeric forms of ParM is necessary for providing a molecular explanation for dynamic instability.

One of the bottlenecks for studying the structure of cytoskeletal proteins that form dynamic filaments using X-ray crystallography is that often heterogeneity due to polymerisation precludes crystallisation. Although there have been instances of protofilaments being observed in the crystal packing (van den Ent et al., 2001; Oliva et al., 2004; Aylett et al., 2010; Matsui et al., 2012), the possibility of obtaining a relevant protofilament structure is rare, especially when the filament is helical with a large repeat distance, as is the case for ParM. Hence, a hybrid approach including helical reconstruction of the filaments using cryo-electron microscopy and subsequent fitting of monomeric crystal structures into the reconstruction is suitable for studying the structures of dynamic cytoskeletal protein filaments. In recent years, technical advances in cryo-electron microscopy have resulted in sub-nanometre resolution reconstructions of filaments (Egelman, 2007; Fujii et al., 2009). Successful examples include actin filaments (Fujii et al., 2010), flagellar polyhooks (Fujii et al., 2009) and needle filaments of the Type III secretion system (Fujii et al., 2012).

In this work, we describe an 8.5 Å cryo-electron microscopy reconstruction of the ParM filament. Previous electron microscopy reconstructions of the ParM filament provided much lower resolution pictures (Orlova et al., 2007; Popp et al., 2008; Galkin et al., 2009). The low resolution of the reconstructions has previously led to many debates about the filament structure, even including the polarity of the protofilaments (Erickson, 2012; Galkin et al., 2012). The subnanometer resolution of the current reconstruction confirms the double-helical, polar structure of ParM filaments. Based on the comparison of the reconstruction with crystal structures of ParM in the monomeric states, we discuss how monomer and nucleotide conformations in the filament state contribute to the dynamic instability of ParM filaments. In addition to the cryoEM reconstruction, the current analysis includes crystal structures of four different conformational states of ParM, thus complementing the existing information about dynamic instability of ParM (Popp et al., 2008; Galkin et al., 2009). These insights are relevant not only to the plasmid partitioning mechanism by ParM, but also relate to questions in other dynamic filament systems such as other bacterial actin-like proteins, F-actin and even microtubules in the eukaryotic cytoskeleton.

The sub-nanometer resolution reconstruction also led to elucidation of the mechanism for plasmid partitioning. Based on the structural data and TIRF (total internal reflection fluorescence) microscopy studies, it was shown that ParR*C* binds to only one end of ParM filaments, and a bipolar spindle of antiparallel ParM filaments drives plasmid segregation (Gayathri et al., 2012). The present work describes a detailed structural analysis of the structures reported in (Gayathri et al., 2012) that leads to a mechanistic explanation for dynamic instability of ParM filaments.

## 2 Materials and methods

### 2.1 Cryo-electron microscopy reconstruction of ParM filaments

**Fig. 2:**
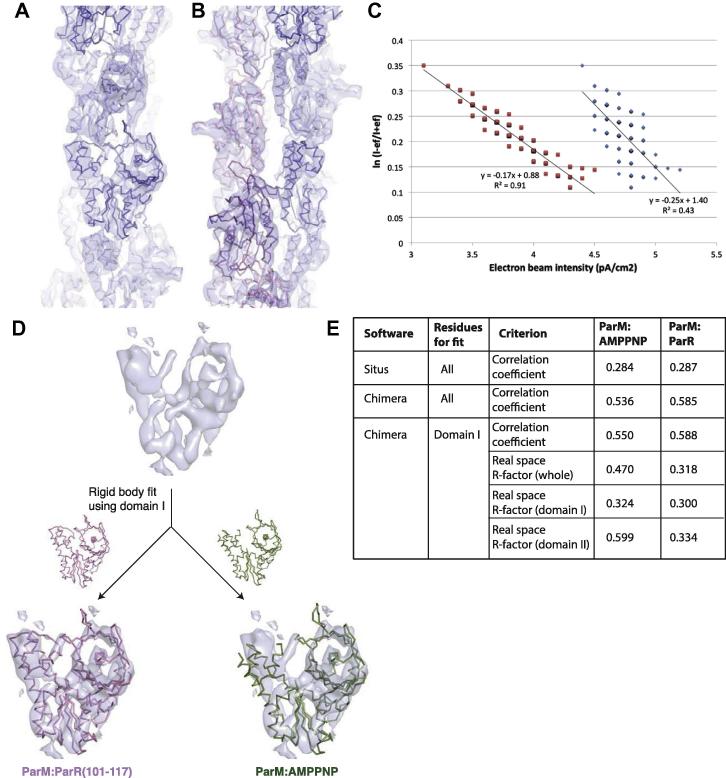
Structure of the ParM filament. (A) and (B) Two views of the cryo-electron microscopy reconstruction of the ParM filament (EMDB ID: EMD-1980). (C) Relationship between the ice thickness and the filtered (red) or unfiltered (blue) intensity. The ice thickness is represented by $\text{ln}\left(\frac{I_{-\mathrm{ef}}}{I_{+\mathrm{ef}}}\right)$ in the ordinate. The distributed points of the filtered and unfiltered intensities over a range of ice thicknesses is fitted by linear regression, respectively. (D) Comparison of the fit of monomeric states of ParM in the EM reconstruction of the filament. ParM-AMPPNP (PDB ID: 4A61) and ParM-AMPPNP with the interacting region of ParR (PDB ID: 4A62) are fitted into a cropped segment of the EM reconstruction corresponding to a single ParM monomer. The table in (E) shows the statistics comparing the fits.

A high-resolution cryoEM reconstruction of the ParM filament (Fig. 2A and B) was obtained as previously reported (Gayathri et al., 2012). Briefly, ParM filaments were prepared by incubating 30 μM ParM protein in 200 μl polymerisation buffer (30 mM Tris–HCl, 25 mM KCl, 2 mM MgCl_2_, 1 mM DTT, pH 7.5) with 5 mM AMPPNP for 5 min at room temperature. The filaments were spun down to remove monomers and resuspended in 40 μl buffer. A 2.1 μl sample solution was applied onto a Quantifoil holey carbon molybdenum grid (R0.6/1.0, Quantifoil Micro Tools GmbH, Jena, Germany) and was plunge-frozen into liquid ethane using a vitrification device (Vitrobot, FEI). The specimen was observed at temperatures of 50–60 K using a JEOL JEM3200FSC electron microscope, equipped with an Ω-type energy filter and operated at 200 kV. Zero energy-loss images, with a slit setting to remove electrons of an energy-loss larger than 10 eV, were recorded on a 4 k × 4 k 15 μM/pixel slow-scan CCD camera, TemCam-F415MP (TVIPS, Germany) at a magnification of 91,463, a defocus range of 0.7–2.0 μM and an electron dose of ∼20 electrons/Å^2^. The magnification was calibrated by the layer line spacing of 23.0 Å of tobacco mosaic virus mixed in the sample solution. The image pixel size at this magnification was 1.64 Å/pixel. 200 CCD images were collected.

Defocus and astigmatism in the images were determined using CTFFIND3 (Mindell and Grigorieff, 2003). Images of the ParM filament were boxed into 20,917 segments of 512 × 512 pixels with a step shift of 100 pixels along the helical axis using EMAN’s boxer program (Ludtke et al., 1999). Images were then phase-corrected by multiplying a phase and amplitude contrast transfer function (CTF) with the astigmatism obtained by CTFFIND3. We used a ratio of 7% for the amplitude CTF to the phase CTF. This procedure results in the multiplication of the square of the CTF (CTF^2^) to the original structure factor and suppresses the noise around the nodes of the CTF, allowing more accurate image alignment. This amplitude modification was corrected in the last stage of image analysis. The images were then high-pass filtered (285 Å), normalised and cropped to 320 × 320 pixels. Image processing was mainly carried out with the SPIDER package (Frank et al., 1996) on a PC cluster computer (RC server Calm2000, Real Computing, Tokyo, Japan).

Projection images were generated from each reference volume at every 1° rotation about the filament axis from 0 to 360° to produce all views. The raw images of the boxed ParM segments were aligned and cross-correlated with the set of reference projections to produce the following information: an in-plane rotation angle, an *x*-shift, a *y*-shift, an azimuthal angle and a cross-correlation coefficient for each segment. Image segments with a small cross-correlation coefficient were discarded. The polarity was tracked and the orientation was determined from the majority for each filament. Image segments identified to have the opposite orientation were discarded. On average, 95% of the segments from each filament showed the same polarity. A 3D reconstruction was then generated by back-projection. The symmetry of this new volume was determined by a least-squares fitting algorithm and was imposed upon the reconstruction (Egelman, 2000). The new volume was used as a reference for the next round of alignment. This process was repeated iteratively until the symmetry values converged. The initial parameters were 24.7 Å for axial rise and 163° for azimuthal rotation along the 1-start helix, and they were converged to 23.62 Å and 164.98°, respectively. The Fourier transform of the reconstruction was then multiplied by 1/[CTF^2^ + 1/SNR] to correct for the amplitude distortion by the CTF. The map was sharpened with a B-factor of −200 Å^2^. The statistics of the EM reconstruction is tabulated in (Gayathri et al., 2012).

### 2.2 Structural analysis

All structure comparisons and superpositions were carried out using PyMOL (Schrödinger Inc.). PDB and EMDB accession numbers involved in the analyses are mentioned in the text and figure legends.

### 2.3 Polymerisation assay for ParM mutants

The mutants described in Fig. 5E were generated using site-directed mutagenesis of plasmid pJSC1 (Salje and Löwe, 2008), and expressed in *E. coli* BL21-AI cells. Cell lysate, from 10 ml cultures after induction with 0.2% arabinose, was used for polymerisation assays of the mutants. 10 mM ATP was added to 200 μl of the cell lysate, and centrifuged at 100,000*g* in a Beckman TLA-100 rotor for 20 min. Pellet and supernatant fractions after centrifugation were loaded onto SDS–PAGE gel, to check for presence of ParM in the pellet fraction. The experiment was repeated with and without nucleotide. The polymerisation assay was performed at expression levels of the mutants and the wild type protein in the lysate. No loss of ParM within inclusion bodies of unfolded protein was observed during the experiment. ParM(L163A) and ParM(D58A) mutants were crystallised in the same space group as the wild type protein, and the crystal structures were determined using ParM(L163A). However, no binding studies with the nucleotide were performed for any of the mutants.

## 3 Results

### 3.1 Structure of the ParM filament at 8.5 Å resolution

A high-resolution cryoEM reconstruction of the ParM filament (Fig. 2A and B) was obtained as previously reported (Gayathri et al., 2012). A key to achieve sub-nanometer resolution in the structural analysis of thin filamentous structures, such as the ParM filament, F-actin and the type III secretion needle, is to obtain cryoEM images of highest possible contrast by making ice layers embedding frozen-hydrated filaments within the holes as thin as possible without physically damaging their structures. An in-column Ω-type energy filter we used for cryoEM image data collection enables the measurement of the ice thickness relatively easily without burning a hole in the ice layer and tilting a grid as usually done otherwise. The thickness of ice (*t*) was estimated from the ratio of the filtered (*I_+ef_*) to the unfiltered intensity (*I_−ef_*) as,

(1) $$\frac{t}{\Lambda }=\text{ln}\left(\frac{I_{-\mathrm{ef}}}{I_{+\mathrm{ef}}}\right)$$

Λ is the mean free path of inelastically-scattered electrons (Egerton and Leapman, 1995). *I*_+_*_ef_* show a strong and clear correlation with the thickness of ice while the correlation between *I*_−_*_ef_* and the ice thickness is much weaker (Fig. 2C). Measuring *I*_+_*_ef_* is a quick way to estimate the ice thickness, and it therefore makes high throughput data collection of high-contrast images possible. In this study, the average value and standard deviation of *I_+ef_* at which we collected data was 74 ± 5% of electron dose exposed (20 electrons/Å^2^). After a few trials of frozen grid preparation with slight variation in conditions, such as the volume of sample solution applied to a holey carbon grid and the blotting time, we were able to find a good grid with the ice layers of appropriate thickness as described above, and collected all 200 CCD image data from this grid in one day.

The energy filter attached to our JOEL electron cryomicroscope (JEM-3200FSC) is very easy to use and reproducible for repeated slit insertion and removal, enabling efficient image data collection routinely. The use of energy filtering for zero-loss imaging also increased the image contrast nearly twofold (Fujii et al., 2009), allowing significantly more accurate image alignment to enhance high-resolution image information. Although the specimen temperature of 50–60 K may be less advantageous for reducing radiation damage than at 4 K, the low-density ice increases the particle image contrast by about twofold in comparison with the high-density ice at 4 K (Fujii et al., 2009). The temperature of 50–60 K also increases the efficiency of high-quality image collection by a few tens fold due to markedly higher heat and electron conductivity than 4 K. All of these factors worked together for the improvement of resolution and throughput of cryoEM structural analysis.

The sub-nanometer resolution of the reconstruction permits clear visualisation of secondary structure elements. It also allows for the unambiguous identification of the best-fitting monomeric structure out of the different conformational states of monomeric ParM (Gayathri et al., 2012), Fig. 2D and E). The model fitting has been carried out with residues of only one of the domains (the fit using Domain I is shown here, using Domain II gives very similar results) using the programs, SITUS (Chacon and Wriggers, 2002) and Chimera (Pettersen et al., 2004). The statistics of the fits are described in terms of correlation coefficients and the real-space R-factors (Fig. 2E). Visual inspection and statistics of the fit (Fig. 2D and E) illustrate that the monomeric conformation of ParM in the crystal structure of ParM, complexed with the interacting region of ParR and AMPPNP (PDB ID: 4A62) fits best into the cryoEM reconstruction. Hence, it is concluded that this conformation closely corresponds to that of the ParM polypeptide within the filament.

There are previous data supporting a heterogeneous nature of monomer conformations within the ParM filament based on low-resolution reconstructions (Galkin et al., 2009). The structural polymorphism within the ParM filament formed in the presence of ATP cannot be ruled out, as the current high-resolution reconstruction was obtained in the presence of AMPPNP. However, the essential nature of many individual interactions at the intra-protofilament interface (see below) suggests that it is improbable that both closed and open states coexist within a stable filament, especially very open conformations. Polymorphism may exist for a transient period of time within an unstable ParM filament, preceding catastrophic disassembly, although it will be difficult to investigate this.

The quasi-equivalence between the filament conformation and the ParR-bound state of ParM has many mechanistic implications (Gayathri et al., 2012). In addition, the best fit provides us with a quasi-atomic view of a ParM monomer within the filament, and thus allows us to speculate upon the conformational changes in the polypeptide chain and the nucleotide during filament formation. This, in turn, provides valuable insights into dynamic instability of ParM filaments. The atomic resolution picture of the filament also (i) provides information on the interfaces of ParM filaments, (ii) allows comparison of different types of actin-like filaments known at atomic resolution (actin, MreB and FtsA), and (iii) helped to identify the complementarity between the surfaces of antiparallel ParM filaments that form the bipolar spindle for plasmid segregation (Gayathri et al., 2012).

### 3.2 Domain movements between ParM conformational states

The conformations of ParM through the complete conformational cycle are currently represented by four crystal structures of ParM: (i) ParM-apo [PDB ID: 1MWK; (van den Ent et al., 2002)], (ii) ParM-ADP [PDB ID: 1MWM; (van den Ent et al., 2002)], (iii) ParM-AMPPNP [PDB ID: 4A61; (Gayathri et al., 2012)], (iv) ParM-AMPPNP with the C-terminal 17 residues of ParR [PDB ID: 4A62; (Gayathri et al., 2012)]. The ParM:ParR bound state (iv) corresponds to the conformation observed in the filament reconstruction (see above), and hence can be approximated as the filament conformation of the monomer. Two additional crystal structures of ParM complexed with GDP and GTP, respectively (Popp et al., 2008), are very similar to states (ii) and (iii) described above, and hence are not included in the discussion.

**Fig. 3:**
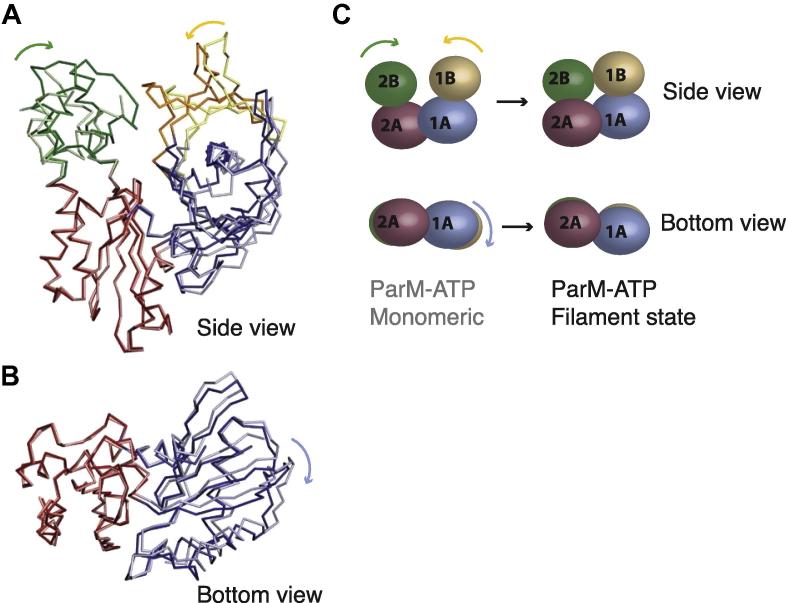
Domain rotations between monomeric and filament states. (A) and (B) Two different views of the superposition of ParM-AMPPNP monomeric state (light shade) and filament state (dark shade). The polypeptides are coloured according to the domains. Domain IIA of the two states are superposed, and the domain movements analysed using DynDom (Hayward and Lee, 2002). (C) A schematic representation of the domain movements.

There is a domain movement of 25° between domains I and II upon nucleotide binding (van den Ent et al., 2002). The two nucleotide bound states, AMPPNP (a non-hydrolysable analogue of ATP) and ADP, corresponding to the pre- and post-hydrolysis states, were found to be quite similar (Gayathri et al., 2012). This somewhat surprising observation could not explain why ParM forms filaments predominantly in the presence of ATP-bound monomers. We can now demonstrate that the conformational change between the monomeric ATP state (ParM-AMPPNP, state (iii)) and the filament state (ParM-AMPPNP: ParR, state (iv)) involves further domain rotations, as depicted in Fig. 3. An analysis of the domain movements using DynDom (Hayward and Lee, 2002) shows 10.7° and 8.9° movements of domains IB and IIB, respectively (Gayathri et al., 2012). There is also an accompanying twist between domains IA and IIA, as shown in Fig. 3B and C. Importantly, similar domain movements, including the twist, have been reported in the transition between G-actin and F-actin (Oda et al., 2009; Fujii et al., 2010). The twist flattens the monomer, creating a perfectly flat surface at the interprotofilament interface of the double-helical ParM filament. This is different from the domain movement within the filament proposed in Galkin et al. (2009), which proposes conformational changes between states that corresponds to the apo and ADP states in the fit of their ParM filament reconstruction.

### 3.3 Nucleotide binding and active site

**Fig. 4:**
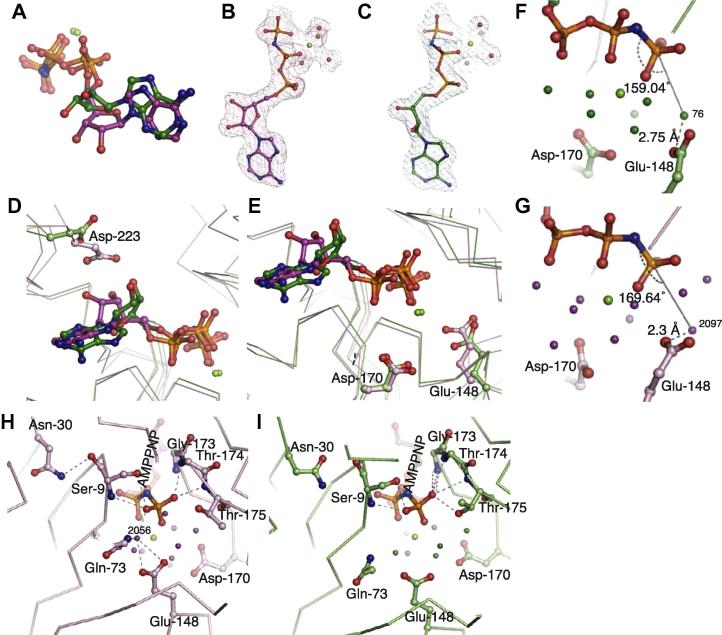
Nucleotide conformation and active site. (A) Comparison of the nucleotide conformations in the monomeric and the filament states. The superposition was obtained by superposing the C-alpha atoms of domain IIA only of the two crystal structures. The nucleotide is shown in ball-and-stick representation, with the C atoms of the monomeric and filament states in green and pink respectively. The ribose ring of the nucleotide in the filament state is in a planar eclipsed conformation, compared to the staggered, 3′ exo conformation of the monomeric state. (B and C) Electron density (2*F*_o_–*F*_c_ at 1σ) for the nucleotide for the two states (B) filament (C) monomeric. (D) Asp-223 of domain IIB of ParM interacts with the hydroxyl groups of the ribose ring in the filament state, holding the ribose in the strained eclipsed conformation. A view of the nucleotide-binding pocket of the superposition of the monomeric (green) and the filament (pink) states are shown. (E) The side chain orientations of the catalytic residues Glu-148 and Asp-170 of ParM are shown. (F and G) A view of the active site, including the waters, is shown for (F) the monomeric state (G) the filament state. The different orientation of the Glu-148 side chain repositions the catalytic water such that it moves towards a linear geometry with respect to the gamma-phosphate. The angles at the phosphorus atom of the gamma-phosphate with respect to the catalytic water, and the distances from the carboxyl group of Glu-148 are highlighted. (H and I) The interactions with the gamma-phosphate for (H) the monomeric state and (I) the filament state are shown. The differences between the two states, which could be coupling factors of interaction with gamma-phosphate and the conformational change, are also highlighted.

The nucleotide binds at the cleft between domains I and II of ParM (van den Ent et al., 2002). The contacts involve residues from all four domains of ParM, which predominantly interact with the ribose and the phosphate moieties of the nucleotide. This most likely explains why ParM accommodates either GTP or ATP in the nucleotide-binding pocket (Popp et al., 2008; Galkin et al., 2009). Asp-223 forms a major interaction with the ribose sugar (Fig. 4D), while the phosphate binding loops consist of residues 9–13 on domain IA (corresponding to P-loop I of actin) and 173–175 of domain IIA (P-loop II of actin). The backbone of residues Ser-9, Gly-173, Thr-174 and Thr-175 form major interactions with the gamma-phosphate (Fig. 4H and I) in the AMPPNP-bound structures (states (iii) and (iv)).

The availability of a crystal structure quasi-equivalent to a filament state for ParM provides a detailed view of the active site geometry within the filament, including catalytic waters, and permits comparison between active sites of monomeric and filament states (Fig. 4A–C). The conformational change in the polypeptide chain is accompanied by a change in the side chain orientation of the catalytic glutamate (Glu-148) of ParM (Fig. 4E). The carboxyl group in the filament state is oriented such that the catalytic water (Wat-2096) is optimally placed with respect to the gamma-phosphate of AMPPNP (Fig. 4F and G). As a result, the angle between the catalytic water and the phosphorous atom of gamma-phosphate is 169°, which is slightly more towards attack geometry (180°), compared to an angle of 159° in the monomeric state (Fig. 4F and G). The carboxyl group of Glu-148 also moves closer to the catalytic water (a distance of 2.3 Å in the filament state compared to 2.75 Å in the monomeric state). We speculate that the differences in the active site geometry favours ATP hydrolysis within the filament state of ParM, as is indeed observed experimentally (Jensen and Gerdes, 1997).

Stimulation of hydrolysis within the filament has also been reported for actin. There are similar arguments for an increased rate of hydrolysis based on the comparison of active site geometries of actin with different metal ions in the monomeric state (Vorobiev et al., 2003). It is highly probable that similar changes, as in the ParM active site also occur in the transition from G-actin to F-actin, leading to stimulation of hydrolysis. The shift of the equivalent Gln-137 and phosphate binding loops towards the nucleotide-binding pocket has been implicated in the activation of hydrolysis within F-actin (Oda et al., 2009; Fujii et al., 2010), although currently no X-ray/atomic resolution structure of the active site of F-actin is available. Of course, in the case of microtubules and FtsZ, the mechanism of stimulation of hydrolysis is different and more intuitive since the incoming subunit directly contributes to the completion of the active site by providing essential residues for catalysis (Nogales et al., 1998).

Surprisingly, comparison of the bound AMPPNP in the crystal structures corresponding to the monomer state and the filament state shows significant differences in the conformation of the nucleotide (Fig. 4A–C). Most notably, the sugar ring of the ribose is held in a planar conformation by Asp-223 of domain IIB of ParM in the filament state (Fig. 4D). The eclipsed conformation of the sugar in the planar ring implies a high-energy conformation of the nucleotide. The oxygens of the three phosphates are also closer to an eclipsed conformation in the filament state compared to the AMPPNP-bound state (Fig. 4B).

Comparison of the contacts made by the gamma-phosphate in the monomeric and filament states (states (iii) and (iv), respectively) shows that the main chain of P-loop I (residues 8–10) moves closer to the gamma-phosphate in the filament state (Fig. 4H and I). This change is also accompanied by a reorientation of the side chain of Asn-30, which putatively couples ATP-binding with conformational change to the filament state. Another additional interaction in the filament state is a water-mediated interaction (Wat-2056) between the gamma-phosphate and Gln-73. We speculate that these interactions, which drive the conformational change to the filament state, do not exist in the ADP-bound state of the monomer, explaining the inability of ParM to polymerise effectively (Garner et al., 2004) and to bind ParR in the presence of ADP (Møller-Jensen et al., 2003; Gayathri et al., 2012).

### 3.4 Filament interfaces of ParM

**Fig. 5:**
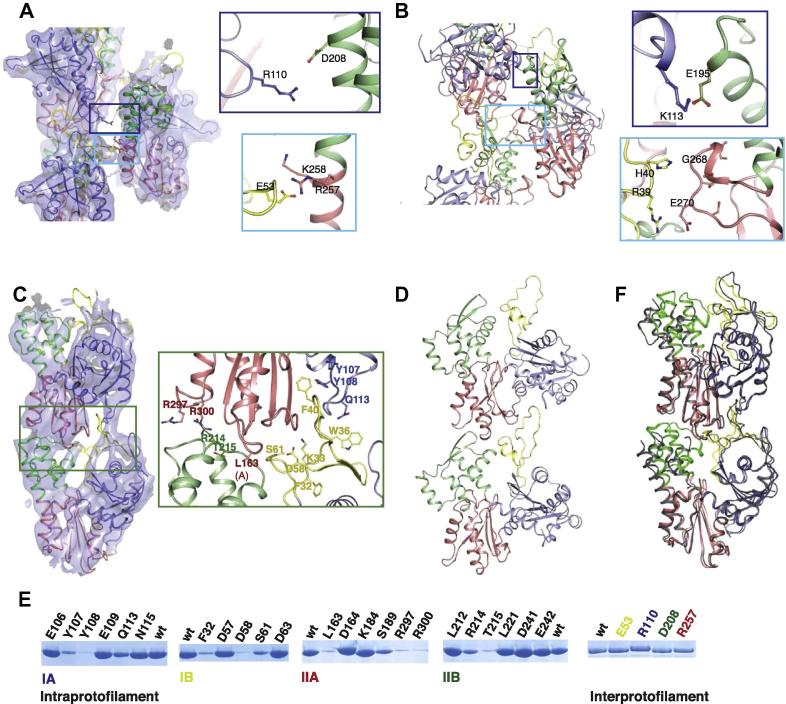
Filament interfaces of ParM and actin. (A) and (B) Inter-protofilament interfaces of ParM (PDB ID: 4A6J) (A) and actin (PDB ID 3MFP) (B). The regions of closest contact are shown in the insets. The corresponding regions in ParM and actin are shown in the same colour of outline for the insets. The cyan-outlined inset corresponds to the hydrophilic plug in actin. The polypeptides are coloured according to the domains. The cryoEM reconstruction of the ParM filament is also shown in surface representation. (C) and (D) Intra-protofilament interfaces of ParM (C) and actin (D). The residues shown to disrupt polymerisation upon mutation (refer (E)) are labelled in the inset for ParM. The cryoEM reconstruction of the ParM filament is also shown in surface representation. (E) Single point mutations at the protofilament interfaces of ParM disrupt polymerisation. Shown are the pellet fractions of the polymerisation assay (Materials and methods) of ParM mutants. All the residues shown have been mutated to alanine. Mutation to alanine of Y107, Y108 of domain IA, F32, D58, S61 of domain IB, L163, S189, R297, R300 of domain IIA, R214, T215 of domain IIB respectively affect polymerisation. (F) A superposition of the ParM-AMPPNP conformation (in grey) on the ParM filament structure (coloured according to the domains) shows that the domains are not optimally positioned to maintain the essential filament contacts.

Fig. 5 shows a comparison of the interfaces between adjacent ParM monomers in the filament with equivalent interfaces of actin. One of the distinguishing features of the inter-protofilament interface in actin is the presence of the hydrophilic plug, which is an insertion at the domain interface of IIA and IIB (equivalent to subdomains 3 and 4) compared to the ParM amino acid sequence. (Though traditionally termed the hydrophobic plug, the interprotofilament interactions at the plug are hydrophilic, and hence was recently renamed as hydrophilic plug (Fujii et al., 2010).) Fig. 5A and B shows that in ParM salt bridges function similar to the hydrophilic plug, at roughly equivalent positions. The interactions between the hydrophilic plug with the DNase-binding loop of domain II in actin has been implicated in slow dynamics at the pointed-end in actin (Narita et al., 2011). Hence, the differences observed in ParM in this region might have implications on the observed differences in filament dynamics, especially regarding actin’s treadmilling versus dynamic instability of ParM. The proposed salt bridges in the inter-protofilament interface of ParM might contribute to the stability of the interface similar to the hydrophilic plug in actin. However, these residues presumably do not hinder the rate of addition of monomers at the pointed-end, leading to an equal rate of growth at both ends for ParM (Garner et al., 2004).

The intra-protofilament interfaces observed in ParM and actin are structurally conserved (Fig. 5C and D), despite ParM forming a left-handed helix as opposed to the right-handed helix of actin. Although the specific residues at the interfaces are not conserved (Galkin et al., 2012), corresponding regions in the structure of ParM and actin are involved in the interactions at the intra-protofilament interface, even if they contain different secondary structural elements. Residues at the interface likely to be essential for polymerisation contacts were mutated to alanine. Many of the single point mutations (Fig. 5E) were sufficient to disrupt the polymerisation. These demonstrate clearly that many crucial contacts involving both subdomain IB and IIB are required for the stability of the filament; and this probably excludes filament models using open conformations of ParM. It also implies that stable filament contacts might be established only by the filament conformation of ParM, and not by the ADP or AMPPNP-bound states of ParM monomer (Fig. 5F).

**Fig. 6:**
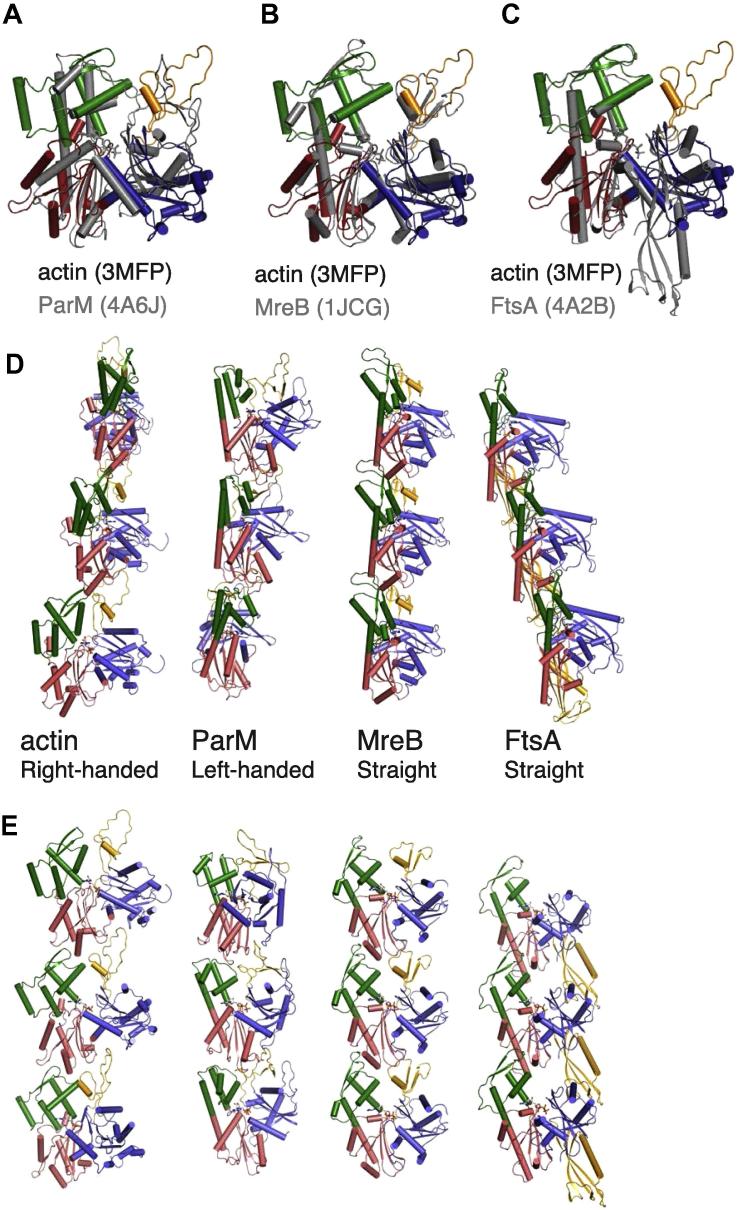
Protofilaments of actin-like fold. (A)–(C) Superposition of actin (domain-wise colours; PDB ID 3MFP) with proteins of the actin-fold (grey), namely ParM [A; PDB ID 4A62], MreB [B; PDB ID 1JCG; (van den Ent et al., 2001)] and FtsA [C; PDB ID 4A2A; (Szwedziak et al., 2012)], with available subnanometer resolution structures of the protofilaments. C-alpha residues of domain IIA (corresponding to subdomain 3 of actin) were used for obtaining the superposition. (D)–(E) Two views of the protofilaments of actin, ParM, MreB and FtsA, with the middle subunit as reference. The superpositions of the middle subunit are shown in (A)–(C). The variation in twist/handedness of the protofilaments despite the similarities between the interfaces is highlighted.

The equivalent interactions at the protofilament interface between left-handed ParM and right-handed actin prompted us to include crystal structures of protofilaments of other actin-like proteins such as MreB and FtsA in the comparison (Fig. 6). In spite of the differences in the specificities of interactions and even in the presence of a domain swap in FtsA, the protofilament architecture is amazingly conserved for the four proteins of actin fold (Fig. 6D and E). This clearly points towards a common evolutionary origin of all filament-forming actin-like proteins. Preliminary characterisation of protofilaments of other Alps (actin-like proteins) such as pSK41 ParM (Popp et al., 2010), pB171 ParM (Rivera et al., 2011) and Alp12A (Popp et al., 2012) also highlights a similar intra-protofilament interface, although high-resolution structures of the filaments are currently not available.

Subtle differences in the interactions at the interface give rise to the differences in the twist observed in the filaments, resulting in a left-handed twist for ParM, right-handed twist for actin and straight protofilaments for FtsA and MreB. It should be noted that all these protofilaments results in a very flat surface on one side, which is involved in the inner/inter-protofilament interface in actin and ParM. The flatness of this surface is attained by the twist of domains I and II upon adopting the filament conformation in ParM (Fig. 3) and for actin (Oda et al., 2009; Fujii et al., 2010). There is evidence that MreB assembles into antiparallel protofilaments such that the membrane-binding sequence of all the MreB monomers in the filament points towards the same surface and the flat protofilament surface faces a perpendicular direction from the membrane (Salje et al., 2011). The twist accompanying the change to the flat conformation has so far not been observed in MreB due to the absence of a crystal structure of MreB in the monomeric state.

## 4 Discussion

### 4.1 Why ParM-ATP forms filaments while ParM-ADP does not

Unlike actin, which can polymerise in the presence of ADP or ATP, the critical concentration for filament formation of ParM in the presence of ADP is about 100 times higher than that of ATP (Garner et al., 2004). The filament presumably disassembles following ATP hydrolysis, in the absence of a cap of ATP-bound monomers, leading to the concept of dynamic instability (Garner et al., 2004). The observation of a conformational change from the ParM-ATP monomeric state (state (iii) described in results) to the filament state (state (iv)) provides a clear rationale for the ATP-dependence of filament formation of ParM, and hence dynamic instability. The filament conformation is essential to form stable filament contacts in ParM. This is highlighted by the observation that single point mutations are sufficient to disrupt ParM polymerisation (Fig. 5E). Many of the essential contacts described in Fig. 5 cannot be formed by the monomeric states of ParM (states (i)–(iii) described in the results section) due to the suboptimal positioning of the interacting residues involved.

We propose that the inability of ParM to polymerise in the presence of ADP is because the transition to the filament conformation is more favourable with ATP than ADP; akin to conformational selection. A comparison of contacts of the nucleotide in the monomeric ADP and AMPPNP states with those in the filament state (Fig. 4H and I) shows that the gamma-phosphate makes many additional contacts with ParM, which in turn, is coupled to the conformational change between the monomeric and filament states of ParM. The changes in Glu-148, Asn-30 and Gln-73 are good examples (Fig. 4H and I). This suggests that the transition to the filament conformation preferentially occurs only in the presence of ATP or AMPPNP. An alternative form in which the filament state has been captured is in the presence of the interacting peptide of ParR, where the interaction with the peptide between subdomains IA and IIA stabilises the filament conformation. The requirement of ATP for binding of ParR (Møller-Jensen et al., 2003; Gayathri et al., 2012) also points towards the dependency on ATP for facilitating the conformational change to the filament state. Interestingly, this dependence on ATP does not exist for actin, as actin can polymerise with ADP.

The conformation of ParM in the filament state, including ATP in the high-energy state, can be described as a meta-stable state and can exist only within the filament, in the presence of lattice constraints, or when bound to ParR. The meta-stable state is stabilised by intra- and inter-protofilament contacts since the subunits are staggered. Importantly, the antiparallel inter-filament pairing of ParM filaments within a bipolar spindle also provides stabilising interactions at the pointed-end, and imposes lattice constraints (Gayathri et al., 2012).

Within the filament, ADP is formed by the activation of ATP hydrolysis and subsequent loss of phosphate. The ADP within the ParM monomers in the filament presumably retains the strained conformation as long as the monomer remains in the filament conformation. Hence, a growing ParM filament (without ParR*C*) can be envisaged to be constituted by ADP-bound monomers flagged on either end by ATP-bound monomers (ATP cap) (Garner et al., 2004). Critically, the length of the ATP cap depends on the difference between the rate of addition of monomers and the rate of ATP hydrolysis within the filament.

### 4.2 Mechanism of dynamic instability

**Fig. 7:**
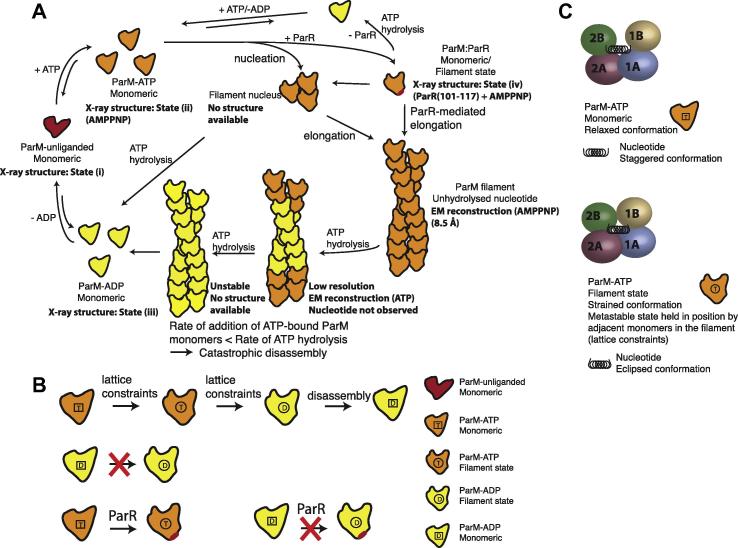
Model for dynamic instability of ParM filaments. (A) A schematic representation of the cycle of events leading to dynamic instability of ParM filaments. The presence of ATP-bound ParM monomers above critical concentration leads to nucleation, followed by elongation of ParM filaments. Filament formation is accompanied by a change in conformation of the ParM monomer to the filament state. This change to the filament conformation is favoured only from the ATP-bound ParM monomer, and not from the ADP-bound ParM monomer, thus explaining the requirement of ATP for filament formation of ParM. ParR-binding to ParM is also accompanied by the conformational change, and hence has a similar dependency for ATP. Once within the filament, ATP hydrolysis is stimulated, due to the optimisation of the active site geometry resulting from the conformational change within the filament. The monomers are held in the filament conformation, as long as there is a constant addition of ATP-bound monomers to the filament ends (maintenance of an ATP cap). Once the pool of ATP-bound monomers is depleted, the ATP cap no longer exists and the filament disassembles following relaxation to the monomeric conformation. (B) The filament state of ParM is a metastable state/strained conformation, which requires filament contacts for its stabilisation. Hence it can exist only within the filament, and forms the basis of dynamic instability. This state can be visualised by an analogy to a clothesline clip. The nucleotide functions analogous to the spring at the centre of the clip.

A summary of events in dynamic instability is depicted schematically in Fig. 7A. As long as ATP-bound ParM monomers are being added at the filament ends, the monomers remain held in the metastable conformational state within the filament, irrespective of hydrolysis and nucleotide state. This corroborates with the proposal of a dynamic instability model of ParM filaments with an ATP cap (Galkin et al., 2009). Even when ATP in ParM subunits within the filament is hydrolysed to ADP, and the phosphate is released irreversibly, the lattice constraints imposed by the filament contacts prevent them from relaxing to the monomeric state. Once there is paucity in ATP-bound ParM monomers, due to the fall in concentration of free ParM-ATP monomers below the steady-state critical concentration, the rate of addition of monomers becomes slower than the hydrolysis rate. Thus, the ATP cap gets depleted, and leads to catastrophic disassembly, due to the absence of additional stabilizing contacts for the terminal subunits. In the presence of excess ATP in the surroundings, the system can enter another cycle of filament formation, since the ParM monomer concentration is now replenished above the critical concentration following disassembly. Thus the cycle of assembly and disassembly continues due to dynamic instability, until all the ATP is hydrolysed.

The model proposed for dynamic instability of ParM filaments can be illustrated by an analogy to a clothesline clip (Fig. 7B). When pressed at the bottom end of the clip, the spring attains a strained state, and the mouth of the clip opens up so that objects can be clasped. Dynamic instability of ParM can be compared to the mechanism of action of the clip. A monomer of ParM in the actin-like fold is similar to the body of the clip with the nucleotide in the centre acting as the spring that holds the two halves (two domains). A grip at the bottom holds the clip and the spring in a metastable state, similar to how ParR is able to induce the conformational change to the metastable filament state of ParM. ParM remains in the metastable state as long as there are lattice contacts holding it. Once the lattice contacts are no longer present, the system relaxes rapidly to its ground state, resulting in depolymerisation or catastrophic disassembly.

It is noteworthy to mention that this model is equivalent to the ‘lattice model’ of microtubules and FtsZ (Oliva et al., 2007; Rice et al., 2008). Even in the presence of GTP, GDP (Matsui et al., 2012) or non-hydrolysable analogues, the change from the curved to the straight conformation occurs only within the filament, when the lattice constraints or the filament contact energy facilitates the transition.
